# Risk factors for dyseugia during chemotherapy in breast cancer women: A cohort STROBE-guided

**DOI:** 10.4317/jced.62680

**Published:** 2026-01-28

**Authors:** Cássia Emanuella Nóbrega Malta, Marcela Maria Fontes Borges, Anna Clara Aragão Matos Carlos, André Alves Crispim, Jennifer Vianna Barbosa, Ana Beatriz Silva Marques Araújo, Lúcio Flávio Gonzaga Silva, José Fernando Bastos de Moura, Paulo Goberlânio de Barros Silva

**Affiliations:** 1 Department of Dental Clinic, Division of Oral Pathology, Faculty of Pharmacy, Dentistry and Nursing, Federal University of Ceará, Fortaleza, Ceará, Brazil; 2 Department of Dentistry, Unichristus, Fortaleza, Ceará, Brazil; 3 Hospital Haroldo Juaçaba, Ceará Cancer Institute, Fortaleza, Ceará, Brazil

## Abstract

**Background:**

To evaluate the taste function of breast cancer patients undergoing Doxorubicin-Cyclophosphamide-Taxanes (AC-T) chemotherapy.

**Material and Methods:**

68 breast cancer patients treated with AC-T were objectively (taste function) and subjectively (scales) evaluated in each chemotherapy cycle. Quality-of-life (QoL), other side effects, clinical-pathological and sociodemographic data, hematological test, general health scores, and the Body Mass Index (BMI) were additionally evaluated. ANOVA-RM/Bonferroni, Friedman/Dunn, chi-square/Fisher's exact tests were applied (SPSS 20.0, p&lt;0.05).

**Results:**

There was a reduction in gustatory sensitivity (p&lt;0.001), salivary flow (p&lt;0.001), periodontal health (p&lt;0.001), and QoL (p&lt;0.001) from the third AC-T cycle and an increase in all side effects. There was an association between low taste sensitivity and lower BMI (p=0.008) and general health scores (p=0.006). Low gustatory sensitivity events was directly associated with the number of AC-T cycles (p&lt;0.001), reduced salivary flow (p=0.019), poorer QoL (p&lt;0.05), and a higher incidence of nausea and anorexia (p&lt;0.05). In addition, age (p&lt;0.001) and trastuzumab (p&lt;0.001) were potential risk factors for dysgeusia.

**Conclusions:**

The incidence of dysgeusia is high in breast cancer patients treated with AC-T; it is cycle-dependent and is associated with weight loss, poorer QoL, and impaired oral and general health. The mechanisms involved in the use of trastuzumab should be investigated.

## Introduction

The modalities available for the treatment of breast cancer depend on the clinical/pathological staging, as well as the phenotype of the tumor, and can be local or systemic treatment (1). The most commonly used modalities are surgery, radiotherapy (RT), chemotherapy (CT), biological therapies, and hormone therapy (1 - 3). There are numerous chemotherapy regimens for breast cancer (4). These drugs are administered intravenously in cycles, which can vary in one or several days of application, and in breast cancer, the interval between cycles is 21 to 28 days (5). The antineoplastic agents used in chemotherapy treatment are toxic to normal, fast-growing tissues, resulting in various adverse effects (6). In Brazil, one of the main chemotherapy regimens is the association of Anthracycline (Doxorubicin) with Alkylating Agents (Cyclophosphamide) followed by Taxanes (paclitaxel and/or docetaxel) for the treatment of breast cancer (5 , 7). Despite their high efficacy in tumor control, the combination of AC and/or T is associated with loss of taste and consequent impairment of quality of life during chemotherapy (8 , 9). Taste function is linked to the lingual taste buds, which depend on the continuous maintenance of taste receptor cells, and the disruption of taste tissue homeostasis caused by chemotherapy drugs is detrimental to the taste system and food intake (10). Taste describes the chemoreception of taste receptor cells in the taste buds, mediated by nerve endings. Taste perception is mediated by specialized neuroepithelial cells structured in organs called taste buds (11 , 12). It occurs through a complex physiological process that includes capturing a stimulus and its transduction (13). Since chemotherapy drugs act by drastically reducing the cellularity of taste buds (13), understanding the risk factors for loss of taste during chemotherapy becomes crucial since dysgeusia can directly affect the food intake of cancer patients, being closely related to worsening nutritional status, increasing cachexia rates, morbidity and mortality, directly affecting the quality of life of these patients (8 , 9). Therefore, this study aims to prospectively evaluate the gustatory function of breast cancer patients treated with TCA and identify potential risk factors for exacerbation of this adverse effect.

## Material and Methods

- Study design and ethical considerations This is a quantitative prospective cohort study using gustatory analysis of breast cancer patients undergoing AC-T chemotherapy protocol without receiving any preventive measures for trans-chemotherapy dysgeusia from April 1, 2021, to April 1, 2023. This project was approved by the Research Ethics Committee of the HHJ/ICC under protocol number 3.286.363 under regulation 466/12 of the Brazilian research ethics legislation, guided by the STROBE. - Inclusion and exclusion criteria Patients over the age of 18 with stage II, III, and IV breast cancer free of previous chemotherapy who had undergone their first adjuvant, neoadjuvant, or palliative treatment with anthracycline-class drug protocols (Doxorubicin (Adrimicin®) alkylating agents (Cyclophosphamide (Cytoxan®) followed by a taxane (Taxol® or Taxotere®) in combination, were selected, and this was referred to as AC-T. Patients with a history of head and neck radiotherapy, smokers, anemia (hemoglobin less than 12 g/dL in women or 13 g/dL in men), untreated diabetes mellitus (glycemia &gt; 200 mg/dL or glycated hemoglobin&gt; 7%), using drugs that significantly alter salivary flow, saliva composition or taste, using centrally-acting analgesics or anxiolytics and antidepressants were excluded. As well as patients with a history of previous dysgeusia or altered sense of smell and taste after COVID-19 (14). All the patients were treated at the chemotherapy outpatient clinic of the HHJ, a High Complexity Oncology Care Center (CACON), during the abovementioned period. Before starting the study, the patients were screened by the multiprofessional team and clinical oncologists in order to select the patients. - Study group and gustatory analysis tool After agreeing to take part in the study and signing the Informed Consent Form clinical-pathological and sociodemographic data were collected, as well as hematological tests (Complete Blood Count and Creatinine), BMI and ECOG, taste function and subjective taste sensation scores. Subsequently, the questionnaire relating to baseline quality of life associated with the oral cavity (OHIP-14) was applied. - Collection of clinical-pathological and sociodemographic data The Electronic Patient Record (EPP) was evaluated to collect clinical and pathological data, including age, co-morbidities, drugs in use, menarche, menopause, parity, nutritional guidelines, psychological support, tumor location, date and type of previous surgical and/or radiotherapy treatments. Tumor characteristics such as pTNM, histological type, immunohistochemistry for hormone receptors (Estrogen Receptor and Progesterone Receptor), HER-2 and ki-67, and sociodemographic data such as race, education, origin, place of birth, family income, and address. Routine pre- and trans-chemotherapy hematological tests, such as complete blood count and renal function markers (creatinine), will be collected via PEP, in the first and last chemotherapy cycles. The data was transcribed into a standard form developed by the team. The body mass index (BMI) and the ECOG scale, used by oncologists to assess the patient's general state of health and the adverse effects reported at each cycle, such as oral mucositis, nausea, vomiting, diarrhea, constipation, anorexia, alopecia, hand and foot syndrome, fatigue, insomnia, and dysuria, will also be collected in the electronic record of patients. Oral health profile and adequacy of the oral environment Before the first chemotherapy session, the patient's oral cavities were inspected by the principal investigator to assess the soft and hard tissues of the maxillo-mandibular complex. The index of decayed, missing, and filled teeth (DMFT) was calculated. Data was collected in the chemotherapy department using a clinical dental photophore attached to the head of the principal investigator. Unstimulated salivary secretion was collected and assessed using the expectoration method. For this method, the individual remained for three minutes without swallowing and, in the end, expelled all the saliva stored in the mouth into a graduated container. 3 ml of saline solution was added so that any droplets of saliva adhered to the wall of the container could decant, and this amount was divided by the number of minutes the patient had spent without swallowing (15). - Assessment of gustatory acuity The objective taste test was carried out before the administration of chemotherapy during four cycles of chemotherapy (AC), followed by a further four cycles (T). For the evaluations of this test, the taste thresholds were carried out with the following substances at the respective molar concentrations (mol/L) and in the following order: sweet (glucose), salty (sodium chloride), sour (citric acid), and bitter (urea) at concentrations of 0.01; 0.032; 0.1; 0.32; 1.0 (0.01- 1.0 mol/L). A single drop of each concentration was applied to the central region of the tongue and swallowed by the patient, starting with the lowest concentration. After applying each drop, the individual evaluated the stimulus for 15 seconds to perceive and identify the taste. If no recognition or identification occurred, the following concentration was applied. Between different flavor modalities, the patient's mouth was rinsed with distilled water (8). To calculate the variation in loss of taste, the -log10 of the concentration of each taste perceived by the patient was calculated, ranging from no change in taste (-log10(0.01) = 2), minimal change in taste (-log10(0.032) = 1.5), moderate change in taste (-log10(0.1) = 1), severe change in taste (-log10(0.32) = 0.5) and severe change in taste (-log10(1) = 0). The sum of the taste loss scores adjusted by -log10, which ranges from 0 to 8, was divided by 8 and multiplied by 100 to obtain the Taste Sensitivity Score (TSS) (%), according to the formula below: [(-log10(sweet)) + (-log10(salty)) + (-log10(sour)) + (-log10(bitter))]*100 / 8. - Subjective taste analysis Patients were also asked during chemotherapy cycles about their subjective perception of taste using a Visual Analog Scale (VAS) scale, ranging from 0 to 10, where 0 corresponds to no change and 10 maximum loss of taste. The CTCAE v3.0 criteria for adverse events was also used, in which the patient classified their taste as (0) no change, (1) change but no change in diet, and (2) change and change in diet; and the Subjective Total Taste Acuity (STTA), a subjective scale of taste acuity that classifies taste as (0) the same as before treatment, (1) slight loss of taste acuity but no inconvenience in daily life, (2) moderate loss of taste acuity and occasional inconvenience in daily life, (3) severe loss of taste and frequent inconvenience in daily life and (4) complete loss of taste acuity (16). These scales were filled in at each cycle (every 21 days) of chemotherapy before the PBMT was applied. - Body mass index (BMI) BMI was calculated on day D0 of the first cycle and on day D0 of the last chemotherapy cycle. The patient was weighed on a conventional scale, and their weight was divided by their height squared to calculate their body mass index (BMI = mass / height²). These data were collected from the ICC's electronic medical records. - Eastern Cooperative Oncology Group (ECOG) Developed in 1982 by the Eastern Cooperative Oncology Group (ECOG) (KARNOFSKY et al., 1982), the ECOG scale is a method of global measurement of the patient's functional performance and an essential therapeutic parameter scale carried out by the oncology team. The patient is classified according to the number of points, ranging from zero (asymptomatic patient, completely active) to four (bedridden patient, requiring more intense care, unable to perform basic self-care) and five (death) (17). - Quality of life analysis The OHIP-14 questionnaire was also applied on day D0 of each cycle from the start of the first chemotherapy cycle (SILVEIRA et al., 2014). The OHIP-14 is a subjective indicator that measures the disability, discomfort, and handicap attributed to the oral condition through self-assessment and its relationship with quality of life. It consists of 14 questions and is a reduced version of the OHIP-49. It is also numbered on a Lickert-type scale with answers ranging from (1) never, 2 (rarely), 3 (sometimes), 4 (repeatedly), and 5 (always). Validated in Brazilian portuguese (18), it is divided into seven domains. The sum of the seven domains makes up the overall quality of life scale (ranging from 14 to 70). - Sample calculation The sample calculation was based on previous study (8), which showed that after four cycles of chemotherapy with doxorubicin and cyclophosphamide, women with breast cancer who underwent photobiomodulation to prevent dysgeusia showed better taste sensitivity (57.82±21.49%) than placebo patients (43.78±23.16%). It was estimated that 55 patients needed to be assessed to obtain a sample with 90% power and 95% confidence. Given the possibility of sample loss, an additional 20% was added to this sample, totaling 68 patients. - Statistical analysis Data on taste sensitivity, quality of life scores, other scales, and quantitative variables were expressed as means and standard deviations and compared using the Wilcoxon or Friedman test, followed by Dunn's post-test. Categorical data was expressed as absolute frequency and percentage and analyzed over the cycles using Pearson's chi-square test. The OHIP-14 questionnaire was subjected to internal consistency analysis in each evaluation period, and Cronbach's coefficient was calculated. After categorizing the loss of taste sensitivity based on the median (50.0%), all the variables were compared with the two categories using the Mann-Whitney, Fisher's exact, or Pearson's chi-square tests and a multiple linear regression model (multivariate analysis).

## Results

- Characterization of the sample The sample consisted of 68 women undergoing antineoplastic treatment for breast cancer using doxorubicin plus cyclophosphamide followed by docetaxel or paclitaxel (AC-T). The mean age of the patients was 50.85±10.75 years, and most were &gt;45 years old (n=46, 67.6%). The average chemotherapy time was 165.43±43.63 days, the average age of menarche and menopause was 13.06±2.41 and 47.41±4.87, and on average, these women had 2.13±1.65 children (Table 1).

Table 1

Almost all the tumors (n=67, 98.5%) were unilateral, and the most frequent staging was T3 (n=25, 39.1%), N1 (n=30, 46.9%) with RE+ (n=51, 75.0%), RP+ (n=45, 66.2%) and ki67&gt;14% (n=48, 70.6%). Only 18 (26.5%) patients were positive for HER2 (3+) (Table 1). The most common intention of chemotherapy was neoadjuvant (n=40, 58.8%) and 56 (82.4%) patients underwent adjuvant radiotherapy. Half of the patients were prescribed adjuvant hormone therapy, and in 19 (27.9%), trastuzumab was used. Four (5.9%) patients had some intercurrence during chemotherapy, and there was no significant variation in the BMI of patients from the beginning (29.82±5.12 to 29.83±5.32 kg/m², p=0.950), with the majority of patients showing a percentage change in mass between losing and gaining 5% of body mass (n=39, 57.4%) (Table 1). Of the 68 patients, 34 received docetaxel and 34 paclitaxel after doxorubicin plus cyclophosphamide. There was no significant reduction in the doses of doxorubicin (p=0.615), cyclophosphamide (p=0.977), docetaxel (p=0.153), or paclitaxel (p=0.735). Creatinine levels (p=0.208) and Neutrophil count (p=0.090) did not vary significantly throughout chemotherapy, but at the end of the eight cycles, there was a significant reduction in hemoglobin (p&lt;0.001), Leukocytes (p=0.004) and Platelet count (p=0.023) (Table 2).

Table 2

**Figure 1:**
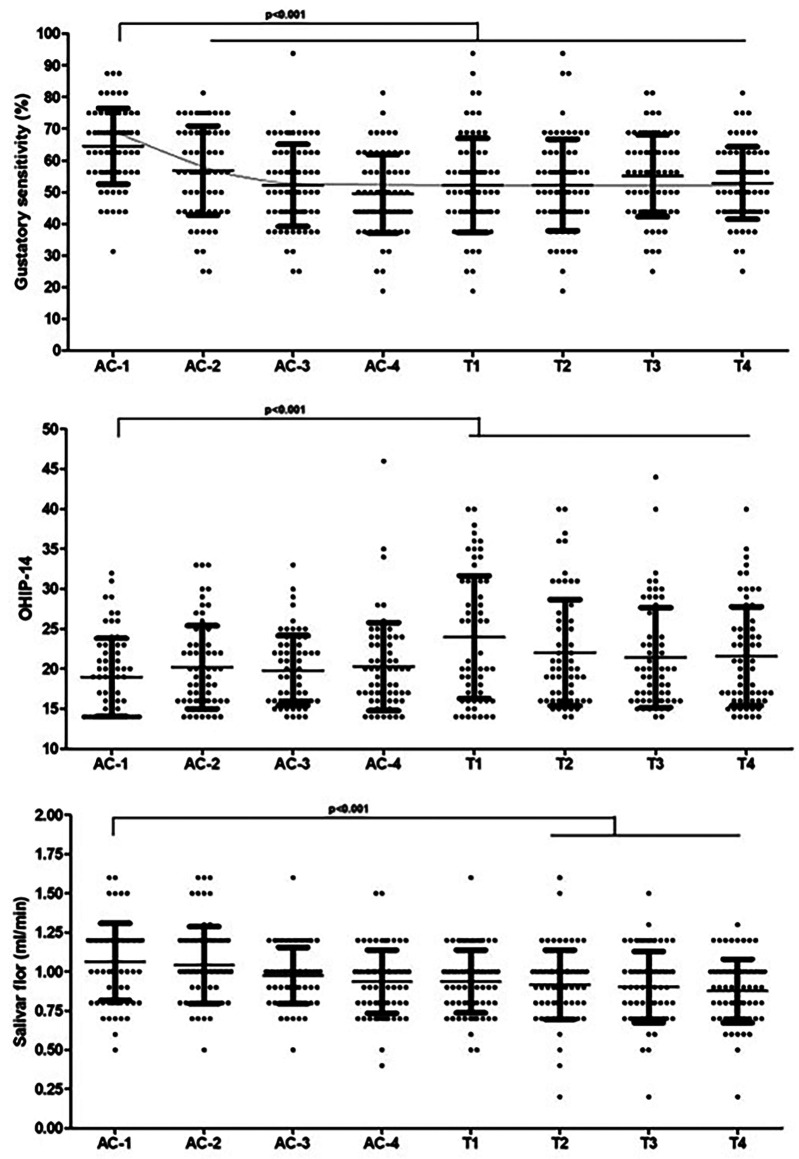
Analysis of taste sensitivity throughout the 4 cycles of AC-T, analysis of the patients’ quality of life and salivary flow throughout the chemotherapy cycles.

- Analysis of taste sensitivity and oral health profile Throughout the eight cycles of chemotherapy, there was a significant reduction in salivary flow, with a significant reduction from the second cycle of taxanes (p&lt;0.001) (Fig. 1).

1

There was a significant reduction in taste sensitivity from AC-2 until the end of chemotherapy (Fig. 1), with a significant reduction during these same periods in sensitivity to sweet (p&lt;0.001&lt; salty (p&lt;0.001) and sour (p&lt;0.001). Only the bitter taste (p=0.205) showed no significant variation throughout the protocol. Both the VAS scale (p&lt;0.001), CTCAE (p&lt;0.001), and STTA (p&lt;0.001) showed an increase from AC-2 until the end of chemotherapy (Table 3).

Table 3

There was no significant variation in the patients' DMFT (p=0.679), but there was a reduction in periodontal health scores from T2 onwards (p&lt;0.001) and an increase in dental mobility scores from T3 onwards (p=0.019) (Table 2). There was a significant increase in all adverse effects in AC-2 (p&lt;0.001) except for dysuria (p=0.336), whose incidence did not vary significantly throughout the study (p=0.336). All adverse effects were significantly reduced throughout taxane administration (p&lt;0.001) (Table 4).

Table 4

- Analysis of oral health-related quality of life The OHIP-14 internal consistency analysis showed adequate Cronbach's alpha values throughout the period. There was a significant increase between AC-2 and T1 in items Q2 (p&lt;0.001), Q3 (p&lt;0.001), Q4 (p=0.002), Q5 (p=0.001), Q6 (p&lt;0.001), Q8 (p=0.005), Q10 (p&lt;0.001), Q11 (p=0.001), Q12 (p&lt;0.001) and Q13 (p&lt;0.001). All OHIP-14 domains increased significantly from T1 onwards (p&lt;0.001) and remained high until the end of the study (Table 5, Fig. 1).

Table 5

- Analysis of predictors of loss of taste sensitivity The 68 patients underwent eight evaluations each, totaling 544 gustatory sensitivity evaluations. The median taste sensitivity score was 50%, with 257 (47.2%) events below this value and 287 (52.8%) events above this value. From the third cycle of chemotherapy onwards, there was a significant increase in loss of taste below 50% (p&lt;0.001). Bilateral tumors (p=0.047), the use of zoledronic acid (p=0.022), and low initial and final BMI (p=0.008) were directly associated with loss of taste below 50% (Table 6). Patients who started chemotherapy with ECOG 1 had lower gustatory sensitivity than patients who started chemotherapy with ECOG 0 (p=0.006), and lower doses of doxorubicin also had lower gustatory sensitivity (p=0.017). Reduced hemoglobin at the start of treatment (p=0.020) and high numbers of neutrophils at the start of treatment (p=0.033) were also significant predictors of loss of taste sensitivity of less than 50%. Reduced salivary flow (p=0.019), DMFT above 21 (p=0.003), nausea (p=0.010), anorexia (p=0.019), and insomnia (p=0.024) were significantly associated with this outcome (Table 6).

Table 6

From a quality of life point of view, questions Q2 (p&lt;0.001), Q3 (p=0.002), Q5 (p&lt;0.001), Q6 (p&lt;0.001), Q9 (p=0.002), Q10 (p=0.002) and Q11 (p&lt;0.001) were significantly affected by the loss of gustatory sensitivity, as were the domains Functional limitation (p&lt;0.001), Psychological discomfort (p&lt;0.001), Psychological disability (p&lt;0.001) and Social disability (p=0.001), resulting in a worse quality of life (p&lt;0.001) (Table 6). In multivariate analysis, age (p&lt;0.001), age at menopause (p&lt;0.001), bilateral location (p&lt;0.001), HER2+ tumor status (p&lt;0.001), use of trastuzumab (p&lt;0.001), high initial creatinine (p=0.006) and high initial neutrophils (p&lt;0.001), development of dysuria (p=0.012) and poor quality of life in the physical pain domain (p=0.013) were independently associated with loss of taste. Increased treatment time (p=0.007), nodal status (p=0.029), number of intercurrences (p&lt;0.001), weight variation (p=0.047), paclitaxel use (p=0.040), high initial leukocytes (p=0.005), high DMFT (p=0.026), and periodontal assessment scores (p=0.019) were associated with better taste sensitivity (Table 6,7).

Table 7

## Discussion

Breast cancer is the most common type of cancer in women, and the combination of anthracyclines, alkylating agents, and taxanes has greatly improved its prognosis in recent decades (19). Although the most severe adverse effects are dose-limiting, dysgeusia, although quite common, does not usually lead to the interruption of cancer treatment (9). This study prospectively assessed taste variation and quality of life in women using TCA and identified important risk factors. The average age of the patients was 50.85±10.75 years old, slightly lower than that described in the literature (20 , 21) and the average treatment time was 165.43±43.63 days, i.e., approximately 5.5 months which conflicts with four cycles of Anthracyclines-Cyclophosphamide every 21 days followed by 4 cycles of Taxanes every 7 or 21 days, paclitaxel or docetacel, respectively (19 , 22 , 23). Most of the samples were T2 tumors or more, with nodal metastasis and hormone-sensitive tumors being the most frequent. Only 18 patients had a HER2+ tumor phenotype, and the proposed complementary therapy for these patients was the inclusion of trastuzumab in the therapeutic regimen, corroborating the profile of breast cancer patients (24 - 26). The effect of the AC-T protocol on the weight variation of the patients evaluated showed no significant change throughout the systemic protocol, suggesting that the multiprofessional follow-up that patients receive at the center is significantly effective since CT negatively affects anthropometric and body composition parameters and can specifically reduce lean mass (27). These findings are important given that weight loss during CT is a risk factor for toxicity and breast cancer recurrence (27 - 29). Despite this, a recent cohort of Danish women did not find an association between pre- and post-chemotherapy weight gain, (30). Since the total dose of the drug is based on the patient's body weight and since no significant change in body mass was identified, there was no change in the dose during the study period due to the maintenance of BMI. Khan (31) describe that the ability to administer the planned dose is vital for tumor control and survival. Neutropenic events are the main limiting factors in achieving this goal (31 - 32). This was not identified during the study since the dose of chemotherapy was not altered during treatment, and only four patients had complications during CT, so there was no significant change in the number of neutrophils despite significant thrombocytopenia. Peripheral blood parameters, such as the neutrophil-lymphocyte ratio (NLR), are prognostic markers for breast cancer patients, as these peripheral blood markers gradually change with the progression of the disease since patients with a high NLR have a poor prognosis (33 - 35). In the present study, it was possible to identify an association, in the multivariate analysis, between a reduction in hemoglobin count and an increase in neutrophils, at the start of antineoplastic treatment, and loss of gustatory sensitivity, which directly interferes with quality of life, general health status and tumor prognosis. This increase in initial neutrophils was an independent factor associated with reduced gustatory function in this study. This showed the strong association of the gustatory profile with hematological balance during cancer treatment and general health status (ECOG) since patients with ECOG 1 had lower gustatory sensitivity. Concerning the oral health profile, a significant reduction in salivary flow could be observed after starting treatment with taxanes (36). Taxanes are neurotoxic drugs and salivary secretion highly depends on parasympathomimetic neurotransmitters (37). In addition, around 96.2% of patients undergoing cancer treatment have oral complications due to hyposalivation (38 , 39). We observed an increase in tooth mobility scores in the last cycles of chemotherapy with taxanes, corroborating the impact of saliva on periodontal health. In the present study, taste sensitivity and oral health-related quality of life worsened after a few cycles of chemotherapy. Taste is extremely important for generating satisfaction during food intake, and the reduction in the gustatory function of breast cancer patients begins soon after the first cycles of chemotherapy (40); the perception of salty taste has been described as the most affected (41), but in the present study sensitivity to sweet, salty, sour and bitter were equally compromised soon after starting CT. This is a common side effect of systemic cancer therapy and negatively impacts the quality of life of cancer patients (8 , 9 , 11). Choi et al. (42) described a strong association between altered taste, xerostomia, and quality of life, findings which were also observed in the present study. A total of 544 evaluations of taste sensitivity were carried out over 8 cycles of chemotherapy, and around half of the patients had a taste sensitivity of less than 50%, with a significant increase in this parameter from the third cycle of CT onwards, which reflects the strong impact of systemic treatment on taste sensitivity. Taste function declines throughout chemotherapy treatment, regardless of the flavors affected, and the longer the treatment, the higher the incidence of dysgeusia, which has a direct impact on nutritional status (8 , 9). Additionally, even though patients did not show significant weight loss, BMI, the ECOG scale and the incidence of nausea and anorexia were inversely associated with gustatory function, reinforcing the association between food intake, general health status and taste (8 , 27 - 29) since lack of appetite decreases food intake and leads to malnutrition and depression (8 , 30 , 43 , 44). In multivariate analysis, additionally, there was an association of low taste sensitivity with the use of Trastuzumab in HER2+ tumors and menopausal status and age. Age is naturally inversely related to loss of taste (8 , 45 , 46), and in a multicenter study of women with HER2+ breast cancer treated with trastuzumab, the frequency of dysgeusia was significantly higher than patients not using this monoclonal antibody (47). However, the mechanisms leading to the impact of trastuzumab use on loss of taste are unknown. Thus, we described in this study that CT leads to dysuria in a manner dependent on the number of cycles, that this effect is associated with xerostomia and interferes with quality of life, incidence of adverse effects, oral health and body mass variation, and that age and use of trastuzumab are potential risk factors.

## Figures and Tables

**Table 1:** Clinical profile of breast cancer patients submitted to taste analysis during chemotherapy with doxorubicin plus cyclophosphamide and taxanes.

	n (%)
Age (years)	50.85±10.75
Up to 45	22 (32.4%)
>45	46 (67.6%)
Treatment time (days)	165.43±43.63
Menarche (years)	13.06±2.41
Menopause (years)	47.41±4.87
Children (n)	2.13±1.65
Location	
Unilateral	67 (98.5%)
Bilateral	1 (1.5%)
T	
T1	5 (7.8%)
T2	23 (35.9%)
T3	25 (39.1%)
T4	11 (17.2%)
N	
N0	16 (25.0%)
N1	30 (46.9%)
N2	16 (25.0%)
N3	2 (3.1%)
Tumor phenotype	
RE+	51 (75.0%)
RP+	45 (66.2%)
HER2+	18 (26.5%)
ki67>14%	48 (70.6%)
Intention QT	
Neoadjuvant	40 (58.8%)
Adjuvant	28 (41.2%)
Adjuvant RT	56 (82.4%)
Adjuvant hormone therapy	34 (50.0%)
Trastuzumab	18 (26.5%)
Zoledronic acid	1 (1.5%)
Intercurrences	4 (5.9%)
BMI (p=0.950) (kg/m²)	
Initial	29.82±5.12
Final	29.83±5.32
Percentage weight change	
< -5%	13 (19.1%)
-5% a +5%	39 (57.4%)
> +5%	16 (23.5%)

Data expressed as absolute frequency and percentage or mean and standard deviation.*p<0.05, Wilcoxon test (mean±SD).

**Table 2:** Hematological tests and doses of antineoplastic drugs in breast cancer patients submitted to taste analysis during chemotherapy with doxorubicin plus cyclophosphamide and taxanes.

	n (%)
Taxane	
Docetaxel	34 (50.0%)
Paclitaxel	34 (50.0%)
Drug dose	
Doxorubicin (p=0.615) (mg)	
Cycle 1	102.53±11.57
Cycle 2	102.11±12.50
Cycle 3	101.16±12.71
Cycle 4	100.96±12.95
Cyclophosphamide (p=0.977) (mg)	
Cycle 1	1003.81±199.31
Cycle 2	1020.37±124.58
Cycle 3	1021.37±158.45
Cycle 4	1021.69±160.77
Docetaxel (p=0.153) (mg)	
Cycle 5	135.38±15.00
Cycle 6	132.47±15.61
Cycle 7	130.96±18.95
Cycle 8	130.83±19.89
Paclitaxel (p=0.735) (mg)	
Cycle 5	135.96±14.87
Cycle 6	136.99±14.97
Cycle 7	136.76±15.11
Cycle 8	136.32±15.21
Hematology tests	
Creatinine (p=0.208) (mg/dl)	
Initial	0.70±0.16
Final	0.66±0.14
Hemoglobin (*p<0.001) (g/dL)	
Initial	12.67±0.95
Final	11.59±1.14
Leukocytes (*p=0.004) (unit/µL)	
Initial	6806±1874
Final	5970±2229
Platelet count (*p=0.023) (unit/µL)	
Initial	326034±100233
Final	298741±87835
Neutrophils (p=0.090) (unit/µL)	
Initial	4366±1669
Final	4131±1730

Data expressed as absolute frequency and percentage or mean and standard deviation.*p<0.05, Friedman test (mean±SD).

**Table 3:** Oral health profile and taste analysis of breast cancer patients undergoing taste analysis during chemotherapy with doxorubicin plus cyclophosphamide and taxanes.

	Chemotherapy cycle								p-Value
	AC-1	AC-2	AC-3	AC-4	T1	T2	T3	T4	
Salivary flow	1.06±0.25	1.04±0.25	0.98±0.18	0.94±0.20	0.94±0.20	0.92±0.22*	0.90±0.23*	0.88±0.20*	<0.001a
Normal	46 (67.6%)*	44 (64.7%)*	40 (58.8%)	37 (54.4%)	33 (48.5%)	33 (48.5%)	30 (44.1%)	31 (45.6%)	0.001 b
Bass	20 (29.4%)	23 (33.8%)*	27 (39.7%)*	27 (39.7%)*	22 (32.4%)	30 (44.1%)	33 (48.5%)	28 (41.2%)	
Very low	2 (2.9%)	1 (1.5%)	1 (1.5%)	4 (5.9%)	13 (19.1%)*	5 (7.4%)*	5 (7.4%)*	9 (13.2%)*	
Quantitative taste evaluation									
Taste sensitivity	64.55±11.99	56.83±14.07*	52.14±12.99*	50.95±17.85*	53.61±19.51*	53.71±19.17*	56.65±17.81*	52.88±11.45*	<0.001 a
Sweet	0.97±0.41	0.79±0.38*	0.68±0.39*	0.60±0.36*	0.71±0.44*	0.66±0.40*	0.65±0.30*	0.62±0.31*	<0.001 a
Salty	1.37±0.35	1.19±0.41*	1.10±0.42*	1.04±0.36*	1.11±0.42*	1.10±0.40*	1.19±0.40*	1.14±0.33*	<0.001 a
Sour	1.88±0.23	1.68±0.39*	1.58±0.33*	1.52±0.36*	1.54±0.32*	1.56±0.35*	1.63±0.34*	1.60±0.33*	<0.001 a
Bitter	0.94±0.50	0.88±0.48	0.81±0.43	0.90±1.18	0.92±1.21	0.98±1.18	1.05±1.18	0.87±0.38	0.205 a
Qualitative taste evaluation									
EVA	0.00±0.00	4.16±2.38*	5.60±1.75*	5.72±2.32*	6.01±2.28*	6.15±2.11*	6.01±1.95*	5.53±2.51*	<0.001 a
CTCAE	0.00±0.00	1.10±0.93*	1.49±0.63*	1.50±0.72*	1.54±0.61*	1.50±0.59*	1.50±0.56*	1.35±0.64*	<0.001 a
STTA	0.00±0.00	1.09±0.81*	1.51±0.68*	1.60±0.96*	1.78±0.77*	1.66±0.86*	1.68±0.78*	1.51±0.95*	<0.001 a
Dental parameters									
CPOD	20.22±8.54	20.34±8.50	20.09±8.52	20.09±8.52	20.09±8.52	20.46±8.52	20.46±8.52	20.46±8.52	0.679 a
Periodontal assessment	0.29±0.52	0.28±0.48	0.28±0.48	0.34±0.51	0.21±0.48	0.16±0.41*	0.21±0.48*	0.12±0.37*	<0.001 a
Tooth mobility	2.24±1.20	2.24±1.19	2.24±1.19	2.18±1.21	2.32±1.21	2.46±1.14	2.51±1.04*	2.60±1.25*	0.019 a

*p<0.05,a Friedman/Dunn test (mean±SD);b Pearson’s chi-square test (n, %)

**Table 4:** Incidence of adverse effects in breast cancer patients undergoing gustatory analysis during chemotherapy with doxorubicin plus cyclophosphamide and taxanes.

	Chemotherapy cycle								p-Value
	AC-1	AC-2	AC-3	AC-4	T1	T2	T3	T4	
Adverse effects									
Mucositis	0 (0.0%)	12 (17.6%)*	17 (25.0%)*	24 (35.3%)*	10 (14.7%)*	8 (11.8%)*	8 (11.8%)*	7 (10.3%)	<0.001
Nausea	0 (0.0%)	58 (85.3%)*	52 (76.5%)*	46 (67.6%)*	40 (58.8%)*	25 (36.8%)*	21 (30.9%)*	14 (20.6%)*	<0.001
Vomit	0 (0.0%)	28 (41.2%)*	18 (26.5%)*	18 (26.5%)*	9 (13.2%)*	5 (7.4%)	13 (19.1%)	3 (4.4%)	<0.001
Diarrhea	0 (0.0%)	9 (13.2%)*	10 (14.7%)*	11 (16.2%)*	5 (7.4%)	18 (26.5%)*	19 (27.9%)*	22 (32.4%)*	<0.001
Constipation	0 (0.0%)	12 (17.6%)*	10 (14.7%)*	14 (20.6%)*	13 (19.1%)*	11 (16.2%)*	10 (14.7%)*	5 (7.4%)	<0.001
Anorexia	0 (0.0%)	37 (54.4%)*	37 (54.4%)*	27 (39.7%)*	4 (5.9%)	7 (10.3%)	7 (10.3%)	5 (7.4%)	<0.001
Alopecia	0 (0.0%)	67 (98.5%)*	54 (79.4%)*	65 (95.6%)*	8 (11.8%)*	14 (20.6%)*	6 (8.8%)	7 (10.3%)	<0.001
Hand-foot syndrome	0 (0.0%)	9 (13.2%)	27 (39.7%)*	11 (16.2%)*	3 (4.4%)	11 (16.2%)*	11 (16.2%)*	21 (30.9%)*	<0.001
Fatigue	0 (0.0%)	44 (64.7%)*	47 (69.1%)*	48 (70.6%)*	29 (42.6%)*	37 (54.4%)*	44 (64.7%)*	32 (47.1%)*	<0.001
Insomnia	0 (0.0%)	19 (27.9%)*	18 (26.5%)*	25 (36.8%)*	3 (4.4%)	14 (20.6%)*	12 (17.6%)*	14 (20.6%)*	<0.001
Dysuria	0 (0.0%)	0 (0.0%)	0 (0.0%)	0 (0.0%)	2 (2.9%)	2 (2.9%)	2 (2.9%)	1 (1.5%)	0.336

*p<0.05, Pearson’s chi-square test (n, %)

**Table 5:** Analysis of oral health-related quality of life in breast cancer patients undergoing taste analysis during chemotherapy with doxorubicin plus cyclophosphamide and taxanes.

	Chemotherapy cycle								p-Value
	AC-1	AC-2	AC-3	AC-4	T1	T2	T3	T4	
Quality of life (items)									
Q1	1.10±0.39	1.06±0.29	1.03±0.17	1.12±0.44	1.10±0.39	1.13±0.57	1.07±0.36	1.15±0.55	0.399
Q2	1.06±0.34	2.54±1.10*	2.65±0.84*	2.63±0.91*	3.12±1.13*	3.03±0.93*	2.91±1.00*	2.75±1.06*	<0.001
Q3	1.21±0.59	1.34±0.75*	1.26±0.51*	1.37±0.69*	1.65±0.91*	1.59±0.92*	1.35±0.73*	1.31±0.63*	<0.001
Q4	1.15±0.53	1.24±0.67	1.25±0.72	1.35±0.84*	1.56±0.97*	1.31±0.65*	1.28±0.62*	1.34±0.70*	0.002
Q5	1.91±1.05	1.85±1.04	1.79±1.07	1.71±0.98	2.10±1.29*	1.87±1.17	1.90±1.15	1.94±1.21	0.001
Q6	1.88±1.06	1.84±1.06	1.88±1.07	1.79±0.99	2.18±1.35*	1.79±1.15	2.00±1.25	2.00±1.25	0.006
Q7	1.21±0.61	1.19±0.55	1.18±0.52	1.26±0.70	1.60±0.95*	1.38±0.79	1.29±0.62	1.34±0.68	<0.001
Q8	1.13±0.57	1.29±1.33	1.10±0.52	1.29±0.81	1.38±0.85*	1.25±0.74	1.09±0.33	1.26±0.66	0.005
Q9	1.54±0.87	1.49±0.82	1.59±0.88	1.50±0.80	1.81±1.10	1.65±1.00	1.50±0.91	1.66±1.09	0.074
Q10	1.25±0.63	1.34±0.64	1.18±0.42	1.26±0.54	1.56±0.84*	1.41±0.70*	1.40±0.74*	1.34±0.59*	<0.001
Q11	1.84±1.03	1.71±1.02	1.62±0.85	1.62±0.86	1.96±1.14*	1.76±1.02	1.84±1.13	1.91±1.13*	0.001
Q12	1.29±0.55	1.13±0.38	1.13±0.34	1.12±0.37	1.41±0.70*	1.35±0.66	1.29±0.69	1.22±0.64	<0.001
Q13	1.29±0.52	1.13±0.34	1.13±0.34	1.21±0.53	1.46±0.78*	1.40±0.69*	1.40±0.69*	1.29±0.60	<0.001
Q14	1.12±0.37	1.06±0.29	1.00±0.10	1.04±0.27	1.09±0.29	1.10±0.31	1.10±0.39	1.07±0.36	0.056
Cronbach's Î±	0.778	0.717	0.725	0.812	0.846	0.836	0.825	0.799	
Quality of life (Domains)									
Functional limitation	2.16±0.56	3.60±1.17*	3.68±0.87*	3.75±0.98*	4.22±1.21*	4.16±1.05*	3.99±1.11*	3.90±1.22*	<0.001
Physical pain	2.35±0.84	2.57±1.07	2.51±0.94	2.72±1.22	3.21±1.61*	2.90±1.28*	2.63±1.12	2.65±1.05	<0.001
Psychological discomfort	3.79±1.91	3.69±1.95	3.68±1.96	3.50±1.79	4.28±2.53*	3.66±2.17	3.90±2.23	3.94±2.30	0.001
Physical disability	2.34±1.07	2.49±1.57	2.28±0.91	2.56±1.40	2.99±1.64*	2.63±1.33	2.38±0.85	2.60±1.16	<0.001
Psychological disability	2.79±1.20	2.82±1.21	2.76±1.13	2.76±1.05	3.37±1.64*	3.06±1.46*	2.90±1.32*	3.00±1.49*	<0.001
Social disability	3.10±1.29	2.84±1.14	2.75±0.95	2.74±1.05	3.37±1.57*	3.12±1.46	3.13±1.52	3.13±1.49	<0.001
Handicap	2.41±0.72	2.19±0.43	2.13±0.34	2.25±0.68	2.54±0.95*	2.50±0.91	2.50±0.92	2.37±0.75	<0.001
OHIP14	18.96±4.88	20.21±5.20	19.79±4.38	20.28±5.51	23.97±7.69	22.03±6.65	21.43±6.26	21.59±6.20	<0.001

*p<0.05, Friedman/Dunn test (mean±SD).

**Table 6:** Influence of clinical profile, dose of antineoplastic drugs, and quality of life in breast cancer patients undergoing gustatory analysis during chemotherapy with doxorubicin plus cyclophosphamide and taxanes.

	Taste sensitivity		p-Value
	Up to 50%	>50%	
Total	257 (47.2%)	287 (52.8%)	-
QT cycle			
1	11 (4.3%)	57 (19.9%)*	<0.001a
2	26 (10.1%)	42 (14.6%)*	
3	37 (14.4%)*	31 (10.8%)	
4	44 (17.1%)*	24 (8.4%)	
5	38 (14.8%)*	30 (10.5%)	
6	37 (14.4%)*	31 (10.8%)	
7	30 (11.7%)*	38 (13.2%)	
8	34 (13.2%)*	34 (11.8%)	
Age			
Up to 45	78 (30.4%)	98 (34.1%)	0.345a
>45	179 (69.6%)	189 (65.9%)	
Treatment time days	163.52±49.29	167.13±37.23	0.108b
Menarche	13.16±2.45	12.97±2.34	0.526b
Menopause	47.65±5.05	47.18±4.61	0.092b
Children	2.20±1.66	2.07±1.62	0.341b
Location			
Unilateral	256 (99.6%)	280 (97.6%)	0.047a
Bilateral	1 (0.4%)	7 (2.4%)	
T			
T1	18 (7.3%)	22 (8.3%)	0.070a
T2	91 (36.8%)	93 (35.1%)	
T3	106 (42.9%)	94 (35.5%)	
T4	32 (13.0%)	56 (21.1%)	
N			
N0	58 (23.5%)	70 (26.4%)	0.799a
N1	116 (47.0%)	124 (46.8%)	
N2	64 (25.9%)	64 (24.2%)	
N3	9 (3.6%)	7 (2.6%)	
Tumor phenotype			
RE+	189 (73.5%)	219 (76.3%)	0.457a
RP+	169 (65.8%)	191 (66.6%)	0.845a
HER2+	68 (26.5%)	76 (26.5%)	0.995a
ki67>14%	180 (70.0%)	204 (71.1%)	0.790a
Intention QT			
Neoadjuvant	149 (58.0%)	171 (59.6%)	0.704a
Adjuvant	108 (42.0%)	116 (40.4%)	
Adjuvant RT	211 (85.8%)	237 (84.0%)	0.580a
Adjuvant hormone therapy	130 (50.6%)	142 (49.5%)	0.797a
Trastuzumab	70 (27.2%)	82 (28.6%)	0.729a
Zoledronic acid	7 (2.7%)	1 (0.3%)	0.022a
Intercurrences	18 (7.0%)	14 (4.9%)	0.203a
Initial BMI	29.25±5.23	30.33±4.91	0.008b
Final BMI	29.25±5.68	30.34±4.86	0.008b
ECOG 1			
0	183 (71.2%)	233 (81.2%)*	0.006a
1	74 (28.8%)*	54 (18.8%)	
2	0 (0.0%)	0 (0.0%)	
ECOG 4			
0	158 (61.5%)	178 (62.0%)	0.982a
1	95 (37.0%)	105 (36.6%)	
2	4 (1.6%)	4 (1.4%)	
Percentage weight change			
<-5%	54 (21.0%)	50 (17.4%)	0.518a
-5% a +5%	146 (56.8%)	166 (57.8%)	
+5%	57 (22.2%)	71 (24.7%)	
Taxane			
Docetaxel	131 (51.0%)	141 (49.1%)	0.668a
Paclitaxel	126 (49.0%)	146 (50.9%)	
Drug Dose			
Doxorubicin (mg)	99.25±14.04	103.56±10.64	0.017b
Cyclophosphamide (mg)	1006.50±174.69	1024.71±152.07	0.063b
Docetaxel (mg)	132.30±16.12	132.52±18.73	0.940b
Paclitaxel (mg)	135.69±17.03	137.65±12.17	0.445b
Initial hematology tests			
Creatinine (mg/dl)	0.70±0.18	0.70±0.15	0.678b
Hemoglobin (g/dL)	12.58±0.95	12.75±0.94	0.020b
Leukocytes (unit/µL)	6945±1820	6681±1893	0.189b
Platelet count (unit/µL)	333975±89769	318923±107276	0.504b
Neutrophils (unit/µL)	4518±1607	4234±1692	0.033b
Salivary flow			
Normal	129 (50.2%)	165 (57.5%)*	0.019a
Reduced	114 (44.4%)*	96 (33.4%)	
Very low	14 (5.4%)	26 (9.1%)	
Dental parameters			
CPOD	21.57±8.22	19.30±8.50	0.003b
Periodontal assessment	0.22±0.44	0.25±0.49	0.720b
Tooth mobility	2.32±1.27	2.37±1.10	0.746b
Adverse effects			
Mucositis	37 (14.4%)	49 (17.1%)	0.393a
Nausea	136 (52.9%)*	120 (41.8%)	0.010a
Vomit	51 (19.8%)	43 (15.0%)	0.134a
Diarrhea	50 (19.5%)	44 (15.3%)	0.204a
Constipation	37 (14.4%)	38 (13.2%)	0.696a
Anorexia	70 (27.2%)*	54 (18.8%)	0.019a
Alopecia	115 (44.7%)	106 (36.9%)	0.064a
Hand-foot syndrome	41 (16.0%)	52 (18.1%)	0.503a
Fatigue	135 (52.5%)	146 (50.9%)	0.699a
Insomnia	60 (23.3%)	45 (15.7%)	0.024a
Dysuria	5 (1.9%)	2 (0.7%)	0.197a
Quality of life (items)			
Q1	1.07±0.35	1.12±0.47	0.192b
Q2	3.02±0.95	2.20±1.12	<0.001b
Q3	1.46±0.75	1.32±0.72	0.002b
Q4	1.28±0.61	1.33±0.82	0.573b
Q5	2.11±1.17	1.68±1.04	<0.001b
Q6	2.21±1.20	1.66±1.04	<0.001b
Q7	1.35±0.70	1.27±0.70	0.055b
Q8	1.23±0.62	1.23±0.90	0.062b
Q9	1.70±0.94	1.49±0.93	0.001b
Q10	1.42±0.69	1.27±0.60	0.001b
Q11	1.93±1.02	1.64±1.02	<0.001b
Q12	1.25±0.55	1.24±0.59	0.664b
Q13	1.33±0.61	1.25±0.57	0.096b
Q14	1.08±0.33	1.07±0.29	0.606b
Quality of life (Domains)			
Functional limitation	4.09±0.99	3.32±1.26	<0.001b
Physical pain	2.74±1.13	2.65±1.22	0.060b
Psychological discomfort	4.33±2.24	3.34±1.88	<0.001b
Physical disability	2.57±1.17	2.50±1.37	0.062b
Psychological disability	3.12±1.33	2.76±1.31	<0.001b
Social disability	3.18±1.30	2.89±1.36	0.001b
Handicap	2.41±0.78	2.32±0.73	0.083b
OHIP14	22.43±5.83	19.78±6.02	<0.001b

*p<0.05, a Chi-square or Fisher’s exact test (n, %),b Mann-Whitney test (mean±SD).

**Table 7:** Multivariate analysis of risk factors predictive of loss of taste sensitivity in breast cancer patients during chemotherapy with doxorubicin plus cyclophosphamide and taxanes.

	p-Value	Î² adjusted	95%CI	
Taste sensitivity				
QT cycle	0.158	-0.092	-1.312	0.215
Age	*<0.001	-1.143	-2.976	-1.040
Treatment time days	*0.007	0.355	0.088	0.539
Menarche	0.322	-0.133	-2.274	0.751
Menopause	*<0.001	-1.019	-4.939	-1.810
Children	0.081	0.386	-0.355	6.139
Location	*<0.001	-0.879	-109.763	-36.443
T	0.624	-0.043	-3.431	2.064
N	*0.029	0.382	0.811	15.069
RE	0.451	-0.240	-28.496	12.713
PR	0.332	-0.293	-25.224	8.561
HER2	*<0.001	-1.041	-47.828	-20.564
ki67	0.347	-0.147	-13.883	4.902
Intention QT	*0.001	0.488	5.858	23.079
Adjuvant RT	0.930	0.010	-8.274	9.043
Adjuvant hormone therapy	0.314	-0.133	-10.806	3.486
Trastuzumab	*<0.001	-0.802	-10.975	-37.969
Zoledronic acid	0.302	-0.128	-30.928	9.631
Trans QT complications	*<0.001	0.423	8.706	28.057
Initial BMI	0.060	7.508	-0.867	40.352
Final BMI	0.089	-7.246	-38.539	2.757
ECOG 1	0.203	0.234	-4.176	19.542
ECOG 4	0.610	0.101	-7.134	12.126
Percentage weight change	*0.047	2.731	0.080	12.857
Taxane	*0.040	0.299	0.391	15.971
Initial creatinine	*0.006	-0.326	-49.928	-8.301
Initial Hb	0.688	0.079	-4.939	7.467
Initial leukocytes	*0.005	1.181	0.003	0.015
Initial platelet count	0.124	0.295	0.000	0.000
Initial neutrophil	*<0.001	-1.932	-0.022	-0.008
salivary flow	0.217	0.077	-2.729	11.933
CPOD	*0.026	0.434	0.088	1.376
periodontal assessment	*0.019	0.194	0.927	10.096
tooth mobility	0.590	-0.035	-1.937	1.104
Mucositis	0.624	-0.030	-5.257	3.161
Nausea	0.626	-0.033	-4.593	2.769
Vomit	0.515	-0.043	-6.651	3.341
Diarrhea	0.813	-0.013	-4.632	3.636
Constipation	0.304	0.066	-2.424	7.726
Anorexia	0.791	-0.021	-5.748	4.385
Alopecia	0.070	-0.130	-7.499	0.302
Hand-foot syndrome	0.693	0.023	-3.195	4.800
Fatigue	0.869	0.011	-3.202	3.787
Insomnia	0.960	-0.004	-4.897	4.653
Dysuria	*0.012	-0.163	-27.663	-3.481
OHIP14 Functional limitation	0.056	-0.198	-4.289	0.055
OHIP14 Physical pain	*0.013	0.217	0.550	4.579
OHIP14 Psychological discomfort	1.000	1.000	0.050	1.521
OHIP14 Physical disability	0.148	0.137	-0.515	3.390
OHIP14 Psychological disability	0.663	-0.057	-3.155	2.010
OHIP14 Social disability	0.057	0.266	-0.074	5.214
OHIP14 Handicap	0.970	0.004	-3.919	4.073
OHIP14	0.154	-0.449	-2.290	0.364

*p<0.05, multiple linear regression.

## Data Availability

The datasets used and/or analyzed during the current study are available from the corresponding author.
